# Genomic characteristics and molecular epidemiology of MRSA from medical centers in Mexico: Results from the Invifar network

**DOI:** 10.1371/journal.pone.0317284

**Published:** 2025-01-27

**Authors:** Gabriela Echaniz-Avilés, Luis Ángel Núñez-García, Eduardo Rodríguez-Noriega, Consuelo Velázquez-Acosta, Luis Esaú López-Jácome, Eduardo López-Gutiérrez, Talia Pérez-Vicelis, Cristina Torres-Báez, Ulises Garza-Ramos, Nadia Rodríguez-Medina, Juan Manuel Barajas-Magallón, Rosario Vázquez-Larios, Aldo Rafael Silva-Gamiño, Rafael Franco-Cendejas, Adolfo Gómez-Quiroz, Elvira Garza-González

**Affiliations:** 1 Centro de Investigación Sobre Enfermedades Infecciosas, Instituto Nacional de Salud Pública, Cuernavaca, Morelos, Mexico; 2 Departamento de Bioquímica y Medicina Molecular, Universidad Autónoma de Nuevo León, Monterrey, Nuevo León, México; 3 Instituto de Patología Infecciosa y Experimental, Centro Universitario de Ciencias de la Salud Universidad de Guadalajara, Guadalajara, Jalisco, México; 4 Instituto Nacional de Cancerología, Ciudad de México, México; 5 Instituto Nacional de Rehabilitación Luis Guillermo Ibarra, Ciudad de México, México; 6 Hospital Regional de Alta Especialidad de Oaxaca, Oaxaca, Oaxaca, México; 7 Hospital Regional de alta especialidad Bicentenario de la independencia, Tultitlán de Mariano Escobedo, Estado de México, México; 8 Hospital General Dr. Miguel Silva, Morelia, Michoacán, México; 9 Departamento de Resistencia Bacteriana, Instituto Nacional de Salud Pública, Cuernavaca, Morelos, México; 10 Laboratorio Dipromi, Morelia, Michoacán, México; 11 Instituto Nacional de Cardiología Ignacio Chávez, Ciudad de México, México; 12 Hospital Angeles de Morelia, Morelia, Michoacán, México; 13 Laboratorio de Microbiología, Hospital civil de Guadalajara, Guadalajara, Jalisco, México; Zhejiang University, CHINA

## Abstract

**Introduction:**

The methicillin-resistant *Staphylococcus aureus* (MRSA) genome varies by geographical location. This study aims to determine the genomic characteristics of MRSA using whole-genome sequencing (WGS) data from medical centers in Mexico and to explore the associations between antimicrobial resistance genes and virulence factors.

**Methods:**

This study included 27 clinical isolates collected from sterile sites at eight centers in Mexico in 2022 and 2023. Antibiotic susceptibility testing was performed using VITEK 2. In addition, WGS was performed using a NovaSeq platform, and a bioinformatic analysis was conducted using several tools.

**Results:**

In this study, 21 strains were CC5, five were CC8, and one was CC93. Moreover, six strains were identified as ST5(CC5)-MRSA-IIa- t895, four strains were found to be ST1011(CC5)-MRSA-IIa-t895, five strains were found to be ST1011(CC5)-MRSA-IIa-t9364, one strain was found to be ST1011(CC5)-MRSA-IIa-t8116, another was found to be ST1011(CC5)-MRSA-IIa-t62, three were found to be ST8(CC8)-MRSA-IVa-t8, one strain was ST5(CC5)-MRSA-IVa-t2, one strain was as ST93(CC93)-MRSA-IVa-t3949, two strains were ST9003(CC8)-MRSA-IVa-t18492, and three strains were ST9034(CC5)-MRSA-V-t2. All SCC*mec* IIa strains showed resistance to levofloxacin and ciprofloxacin, and all but two strains were resistant to clindamycin. Among the strains that harbored the type IIa cassette, most had the *aadD*, *blaZ*, and *ermA_SDS* genes and the *erm A* gene. Multiple genes for adhesion, enzymes, immune evasion, and secretion system were detected, regardless of SCC*mec* type. Of the SCC*mec* IVa strains, most harbored the Panton-Valentine leukocidin encoding genes.

**Conclusion:**

In this study, the most frequently detected CC was CC5, followed by CC8, and CC93, and the most frequently detected MRSA ST was ST1011, followed by ST5. Most SCC*mec* elements were found to be type IIa, followed by type IVa. High MIC values were observed for ciprofloxacin, erythromycin, and clindamycin, particularly within SCC*mec* IIa. Of the SCC*mec* IVa strains, most harbored the *lukS*-PV and *lukF*-PV genes.

## Introduction

*Staphylococcus aureus* is an important human pathogen and can cause several diseases [1,2], including bacteremia [3]. As such, *S. aureus* contains a diversity of virulence factors and toxins that favor the development of disease but also allow *S. aureus* to address challenges presented by the human immune system [4,5].

*S. aureus* has a high level of resistance acquisition against multiple antibiotic classes, which complicates the treatment of infections associated with this bacterial species [6,7]. Penicillin-resistant epidemic *S. aureus* strains were followed by the so-called “archaic” methicillin-resistant *S. aureus* (MRSA) strains first found in the United Kingdom. In the 1980s, novel lineages emerged and have now achieved worldwide distribution [4]. MRSA strains produce an altered penicillin-binding protein, which is encoded by the *mecA* gene and carried within a mobile genetic element designated staphylococcal cassette chromosome *mec* (SCC*mec*) [8].

The Panton-Valentine leukocidin (PVL) is a widely studied toxin, has been associated with neutrophil lysis by the activity of a two component toxin (LukS-PV and LukF-PV) that assemble on neutrophil membranes, leading to lysis. The role for PVL in pathogenesis is unclear [2].

Several methods have been used to describe MRSA lineages, including pulsed-field gel electrophoresis (PFGE) [9], multilocus sequence typing (MLST), *spa* typing, and SCC*mec* typing [10]. The information obtained from these methods can be useful in tracing outbreaks. Advances in whole genome sequencing (WGS) technologies allow deeper understanding virulence factors and antimicrobial resistance [11], becoming the method of choice for outbreak analysis of microbial pathogens, including MRSA [11,12].

The use of genomic approaches to develop a genome-wide, gene-by-gene analysis tool includes extended MLST (eMLST) that involves ribosomal MLST (rMLST), core genome MLST (cgMLST), whole-genome MLST (wgMLST), and a pan-genome approach [12,13].

The antimicrobial resistance of *S. aureus* has significantly varied over time and by geographical location, which highlights the importance and clinical relevance of molecular surveillance for MRSA [14]. Therefore, our study aims to determine the genomic characteristics of MRSA using whole-genome sequencing (WGS) data from medical centers in Mexico and to explore the associations between antimicrobial resistance genes and virulence factors.

## Materials and methods

### Study design and analysis

This study encompassed 27 MRSA clinical isolates, gathered from eight different centers in Mexico between July 1st, 2022, and June 30, 2023. Clinical isolates were sent to the coordinating laboratory for susceptibility tests and WGS on August 30, 2023. Each microbiology laboratory processed clinical specimens and recovered and identified the strains using conventional methods. All identifications were confirmed using MALDI-TOF.

Twenty clinical isolates were recovered from blood; one from each of the following sources: cerebrospinal, ascites, pericardiac, peritoneal, and synovial fluids; one from soft tissue; and one from a skin abscess.

All participating centers belonged to a network called Red Temática de Investigación y Vigilancia de la Farmacorresistencia (Spanish acronym: INVIFAR).

Data from clinical isolates included antibiotic susceptibility testing, which was performed using VITEK2 (bioMérieux, Marcy l’Etoile, France), disk diffusion method and interpreted based on Clinical and Laboratory Standards Institute (CLSI) criteria [15]. MRSA was defined as *S aureus* with a zone of inhibition ≤21 mm for cefoxitin on Mueller Hinton agar after 16–18 hours of incubation [15].

### Whole-genome sequencing and genome assembly

WGS was performed at Novogene Inc. (Sacramento, CA) on a NovaSeq platform (Illumina Inc., San Diego, CA) using 150-bp paired-end chemistry. *De novo* assembly was completed using Unicycler v0.4.9b (https://github.com/rrwick/Unicycler); contigs <200 bp were discarded. Pathogenwatch online platform [16] was used to obtain genome assembly quality statistics, which are provided in S1 Table.

### Data analysis

SCC*mec* and *spa* typing were performed using the SCC*mec*Finder 1.2 (https://cge.food.dtu.dk/services/SCC*mec*Finder/) tool and the Ridom SeqSphere+ Software v9.0, respectively. For virulence gene screening, draft genomes were analyzed using the Virulence Factor Database’s VFanalyzer tool (http://www.mgc.ac.cn/VFs/main.htm). Antimicrobial resistance gene prediction and MLST were conducted using the Pathogenwatch platform. Raw sequencing reads were mapped to the *S. aureus* NCTC 8325 reference genome (GenBank accession GCA_000013425.1) using Snippy v4.6.0 (https://github.com/tseemann/snippy) for variant calling. Single nucleotide polymorphism (SNP) alignments were generated using the snippy-multi function and used for maximum-likelihood phylogenetic reconstruction with RAxML v8.2.12 (https://github.com/amkozlov/raxml-ng), employing the GTRCAT model of nucleotide substitution. Phylogenetic trees were exported in Newick format and visualized with iTOL v6.5.2 (https://itol.embl.de/).

To compare the sequenced isolates with publicly available genomes, assemblies from the NCBI RefSeq database were downloaded and processed using sourmash v4.8.9 [17] to generate DNA signatures and a signature database. Sourmash uses k-mer based methods to generate “sketches,” which are compressed representations of genome sequences that allow for rapid comparison of large datasets. The 20 most similar genomes for each of the sequenced isolates in this study were identified using the sourmash –compare function, which compares the generated signatures to quantify the similarity between genomes. The final list of RefSeq genomes was filtered to remove duplicate assemblies, retaining only one genome per BioProject ID per year. An exception was made for genomes isolated from sterile sites, in which case more than one isolate per year was included.

Assemblies were annotated using Prokka v1.14.5 [18] and analyzed with Roary v3.13 [19] with the flag –e to generate a core genome alignment using PRANK [20]. The core genome alignment was then introduced to RAxML v8.2.12 [21] with the flags –p 1234, –m GTRCAT to generate a midpoint rooted phylogenetic tree. The presence of *mecB* and *mecC* genes was screened using BLAST+ [22], and MLST was determined using the MLST command line tool (Seemann T, mlst Github https://github.com/tseemann/mlst). Results were integrated and viewed using iTOL v6 [23].

### Statistical analysis

Antibiotic resistance among clonal complexes was compared using the chi-square test with Yates correction when appropriate. Statistical analysis was performed using the SPSS software v 26.

### Ethics approval

The local ethics committee of Hospital Civil de Guadalajara Fray Antonio Alcalde (Jalisco, Mexico) approved this study (Reference No. 29/22). The ethics committee waived informed consent, and all participating institutions agreed with the present study.

## Results

### Clinical isolates and susceptibility results

In total, 27 strains were included in this study; most of them were collected from blood (*n* = 21). All strains were cefoxitin resistant. High MIC values were observed for ciprofloxacin, erythromycin, and clindamycin. In particular, all SCC*mec* IIa strains showed resistance to levofloxacin and ciprofloxacin, and all but two showed resistance to clindamycin. Complete results for the susceptibility tests are shown in Table 1

**Table 1:** Distribution of the SCC*mec* type, MLST, susceptibility tests among MRSA strains included.

Strain	CC	SCC*mec*	*spa* type	ST	Specimen	CFT	CIP	LVX	ERY	CLI	LNZ	DAP	VAN	TET	NIT	RIF	SXT
23-2979	5	IIa	895	5	Blood	1	≥8	≥8	≥8	>4	1	0.5	1	≤1	32	≤0.03	≤10
23-2959	5	IIa	895	5	Blood	≥8	≥8	≥8	≥4	1	0.25	≤0.25	≤0.5	≤1	32	≤0.03	≤10
22-1697	5	IIa	895	5	Blood	1	≥8	≥8	4	≤0.12	2	0.25	≤0.5	≤1	≤16	≤0.03	≤10
22-1714	5	IIa	895	5	Blood	0.5	≥8	≥8	≥8	≥4	1	0.25	≤0.5	≤1	≤16	≤0.03	≤10
23-2905	5	IIa	895	5	CSF	0.5	1	0.5	1	≥4	1	0.25	≤0.5	≤1	≤16	≤0.03	≤10
23-2438	5	IIa	895	5	Peritoneal fluid	0.5	≥8	≥8	≥8	≥4	1	0.25	≤0.5	≤1	≤16	≤0.03	≤10
22-1364	5	IIa	895	1011	Blood	0.5	≥8	≥8	≥8	≥4	1	0.25	≤0.5	≤1	≤16	≤0.03	≤10
23-2539	5	IIa	895	1011	Blood	1	≥8	≥8	≥8	≥4	2	0.5	≤0.5	≤1	≤16	≤0.03	≤10
23-2537	5	IIa	895	1011	Blood	ND	≥8	≥8	≥8	≥4	2	0.5	1	≤1	≤16	≤0.03	≤10
23-2538	5	IIa	895	1011	Blood	0.5	≥8	≥8	≥8	≥4	2	0.25	1	≤1	≤16	≤0.03	≤10
23-2388	5	IIa	9364	1011	Blood	ND	≥8	≥8	≥8	≥4	2	0.5	≤0.5	≤1	≤16	≤0.03	≤10
23-2540	5	IIa	9364	1011	Blood	ND	≥8	≥8	≥8	≥4	ND	≥8	ND	≤1	≤16	≤0.03	≤10
23-2574	5	IIa	9364	1011	Blood	ND	≥8	≥8	≥8	≥4	2	2	ND	≤1	≤16	≤0.03	≤10
23-2575	5	IIa	9364	1011	Blood	ND	≥8	≥8	≥8	≥4	2	0.5	≤0.5	≤1	≤16	≤0.03	≤10
23-2940	5	IIa	9364	1011	Blood	1	≥8	≥8	≥8	≥4	2	0.25	≤0.5	≤1	32	≤0.03	≤10
23-2201	5	IIa	8116	1011	Blood	1	≥8	≥8	≥8	≥4	2	0.25	1	≤1	≤16	≥4	≤10
22-1752	5	IIa	62	1011	Soft tissue	ND	≥8	≥8	≥8	≥4	1	0.25	ND	≤1	≤16	≤0.03	≤10
23-2511	8	IVa	8	8	Blood	0.25	1	0.5	≥8	≤0.12	1	0.25	≤0.5	≤1	≤16	≤0.03	≤10
23-2229	8	IVa	8	8	Blood	ND	≥8	4	≥8	ND	2	0.5	≤0.5	≤1	≤16	≤0.03	≥320
23-2228	8	IVa	8	8	Blood	ND	≥8	4	≤0.25	ND	2	0.5	1	≤1	≤16	≤0.03	≥320
22-1695	5	IVa	2	5	Blood	ND	≤0.5	≤0.12	≤0.25	ND	≤1	0.5	ND	≤1	≤16	≤0.03	≤10
23-2165	93	IVa	3949	93	Pericardiac fluid	ND	≤0.5	≤0.12	≥8	≥4	1	0.25	≤0.5	≤1	≤16	≤0.03	≤10
22-1243	8	IVa	18492	9003	Blood	0.25	≥8	4	≥8	≥4	1	0.25	≤0.5	≤1	≤16	≤0.03	≤10
22-1232	8	IVa	18492	9003	Blood	ND	≥8	4	≥8	ND	1	0.5	≤0.5	≤1	≤16	≤0.03	≤10
23-2580	5	V	2	9034	Synovial fluid	0.25	1	0.5	≤0.25	≤0.12	2	0.25	≤0.5	≥16	≤16	≤0.03	≤10
23-2619	5	V	2	9034	Ascites fluid	1	≥8	≥8	≥8	≥4	1	0.25	1	≤1	≤16	≤0.03	≤10
23-2321	5	V	2	9034	Blood	ND	2	1	4	0.25	2	0.5	≤0.5	≤1	≤16	≤0.03	≤10

Ceftaroline = CFT; ciprofloxacin = CIP; levofloxacin = LVX; erythromycin = ERY; clindamycin = CLI; linezolid = LNX; daptomycin = DAP; vancomycin = VAN; tetracycline = TET; nitrofurantoin = NIT; rifampicin = RIF; trimetoprim-sulfamethoxazole = SXT; cerebrospinal fluid = CSF. All strains were cefoxitin-resistant and had a MIC ≥ 0.5 for benzylpenicillin. No data = ND. Clonal complex = CC. Sequence type = ST.

### Clonal complexes, MLST profiling, SCC*mec*, and *spa* typing

**Fig 1:**
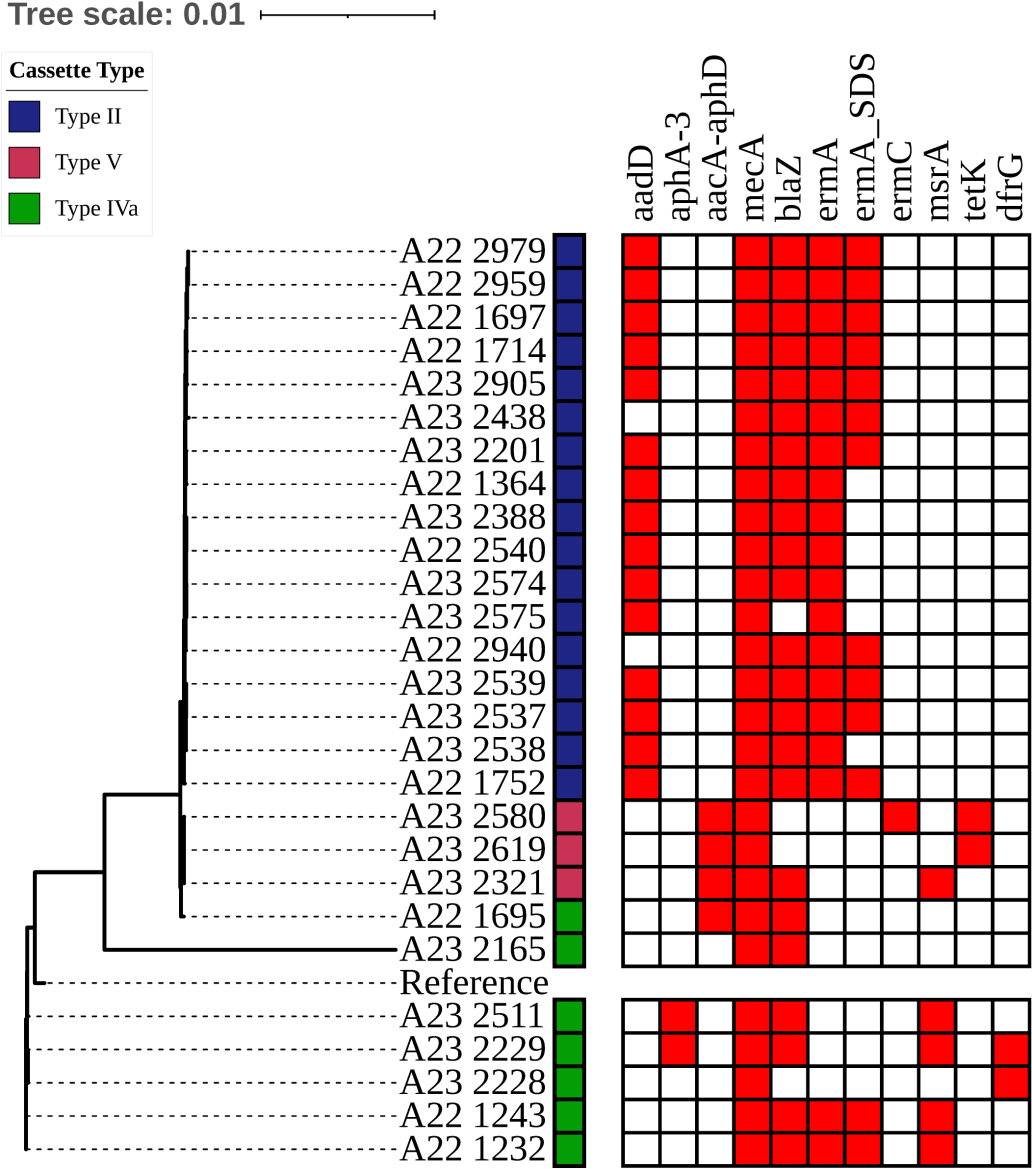
Whole genome midpoint-rooted SNP phylogenetic tree and antimicrobial resistance-associated genes identified by Pathogenwatch. The colors to the right of the sample names depict the type of cassette predicted by the SCC*mec* finder platform.

The most frequent SCC*mec* detected was IIa (*n* = 17); all of these were CC5. Moreover, two different STs were detected: ST5 (*n* = 7) and ST1011 (*n* = 11), followed by Type IVa (*n* = 7) and Type V (*n* = 3; Table 1, Fig 1).

Most strains belonged to common and non-overlapping CC; the most important was CC5 (*n* = 18), which included strains with ST1011 (*n* = 11), followed by ST5 (*n* = 7) and SCC*mec* types IIa (*n* = 17) and IVa (*n* = 1; Table 1). They were followed by CC8 with ST8 (*n* = 3). One strain from CC93 with ST93, and five strains harbored undescribed STs.

Antibiotic results were compared among CC detected. The MIC (Minimum inhibitory concentration) value of ≥8 for levofloxacin was most observed in clonal complex 5 (CC5) compared to other clonal complexes, with a statistically significant p-value of 0.002. No other significant differences were found between the CCs.

### Clones detected

Six strains were found to be ST5(CC5)-MRSA-IIa-t895, four strains were found to be ST1011(CC5)-MRSA-IIa-t895, five strains were ST1011(CC5)-MRSA-IIa-t9364, one strain was ST1011(CC5)-MRSA-IIa-t8116, and other was ST1011(CC5)-MRSA-IIa-t62. Three typed ST8(CC8)-MRSA-IVa-t8, one ST5(CC5)-MRSA-IVa-t2, one was ST93(CC93)-MRSA-IVa-t3949, two were ST9003(CC8)-MRSA-IVa-t18492, and three strains were ST9034(CC5)-MRSA-V-t2. Quality metrics obtained from genome assembly are shown in S1 Table.

### Distribution of SCC cassette type and antimicrobial resistance-associated genes

Among the strains that harbored the type IIa cassette, most had the *aadD*, *blaZ*, *ermA* and *ermA*_SDS genes (the *ermA*_SDS feature indicating variants in the *ermA* gene associated with resistance to clindamycin, in addition to erythromycin). Among the strains that harbored the IVa cassette, some had the *aphA-3*, *blaZ*, *ermA*, *ermA*_SDS, *msrA*, and *dfrG* genes. Only three strains had the type V cassette; all three strains had the *aacA-aphD* gene; and some had the *blaZ*, *ermC msrA,* and *tetK* genes (see Fig 1).

### Distribution of SCC*mec* type and predicted antimicrobial susceptibility patterns

**Fig 2:**
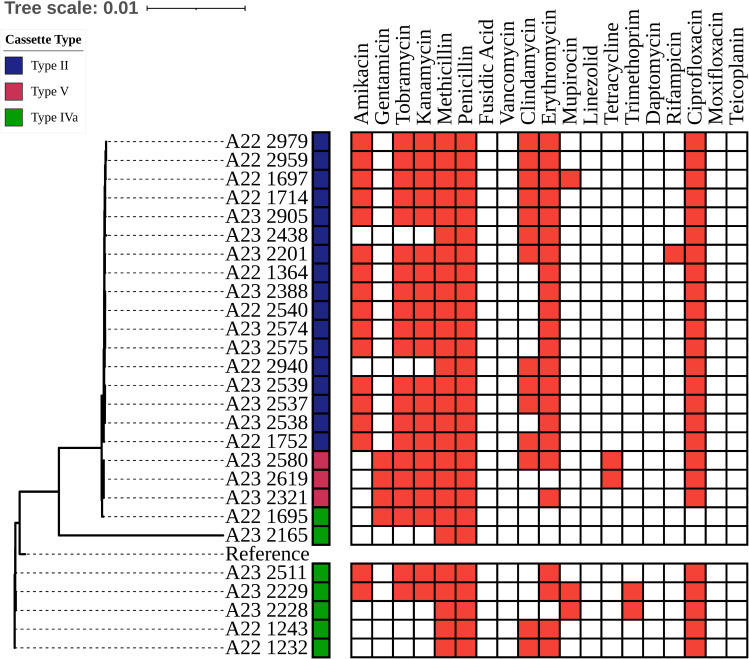
Whole genome midpoint-rooted single nucleotide polymorphism phylogenetic tree and antimicrobial susceptibility patterns predicted by Pathogenwatch. The colors to the right of the sample names depict the type of cassette predicted by the SCC*mec* finder platform.

In all strains, no resistance was predicted for fusidic acid, vancomycin, linezolid, daptomycin, moxifloxacin, and teicoplanin. Resistance was predicted for most strains of amikacin, tobramycin kanamycin, clindamycin, erythromycin, and ciprofloxacin (see Fig 2).

### Whole genome SNP phylogenetic tree and virulence genes

**Fig 3:**
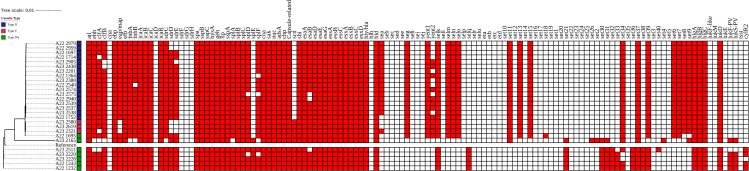
Whole genome midpoint-rooted single nucleotide polymorphism phylogenetic tree and virulence factors predicted by Pathogenwatch. The colors to the right of the sample names depict the type of cassette predicted by SCC*mec* finder platform.

The whole genome SNP phylogenetic tree and virulence factors (adhesion, enzymes, immune evasion, secretion systems, and toxins) detected are shown in Fig 3. Multiple genes for adhesion, enzymes, immune evasion, and secretion system were detected, regardless of SCC*mec* type, including *fmbA*, *fmbB*, *icaA*, *icaB,* and *icaC* (adhesion); *lop* and *nuc* (enzymes); capsule-related (immune evasion); and *esaA, esaB, and esaD* (secretion systems).

By contrast, the detection of toxins showed differences according to SCC*mec*, with high similarity among strains that harbored the IIa and V SCC*mec* (both SCC*mec* harbored several genes, including *seg*, *selm*, *seln,* and *selo*). The Panton-Valentine leukocidin encoding genes (*lukS*-PV y *lukF*-PV) were detected among SCC*mec* IVa strains.

### Comparison with RefSeq genomes

A total of 16,393 genomes were retrieved from the NCBI RefSeq assembly database. After processing with sourmash v4.8.9 [17], 206 genomes (without duplicates) were selected for further analysis. Additional filtering kept only one isolate per BioProject per year, except for samples isolated from sterile sites, where more than one isolate was included. This resulted in a final sample set of eighty isolates (metadata included in S2 Table).

**Fig 4:**
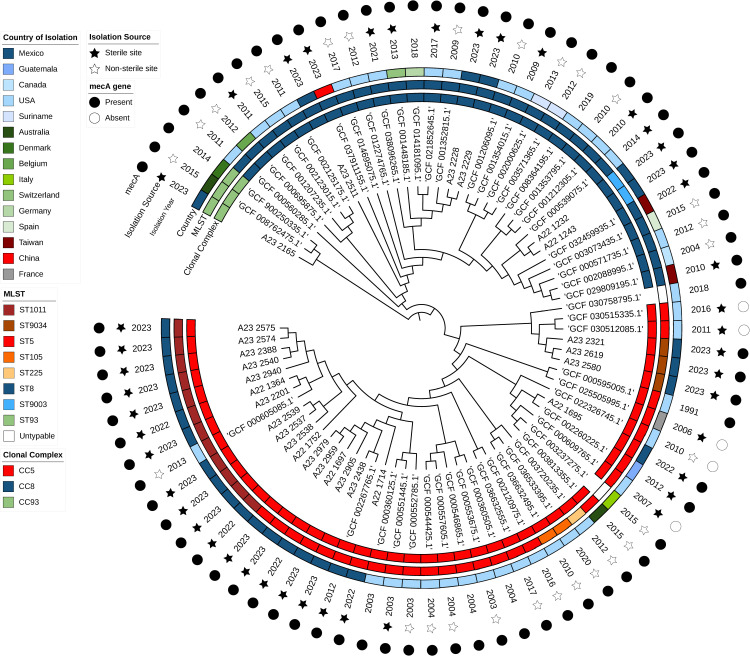
Core genome alignment-based maximum likelihood phylogenetic tree of sequenced MRSA isolates and RefSeq similar genomes.

**Fig 5:**
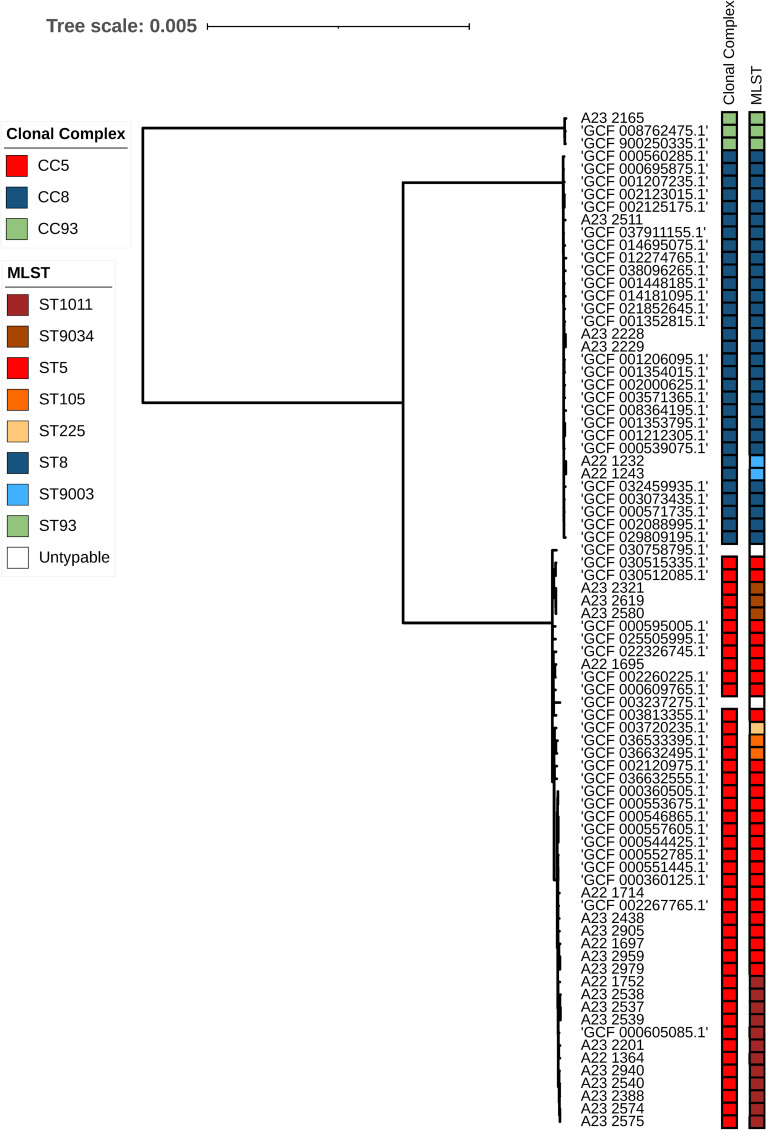
Core genome alignment-based maximum likelihood phylogenetic tree of sequenced MRSA isolates and RefSeq similar genomes with branch distances. The tree scale value represents substitutions per site.

Among these isolates, three different clonal complexes were identified; CC5 was the most frequent [n = 46, STs; 5 (n = 26), 1011 (n = 12), 9034 (n = 3), 105 (n = 2), 225 (n = 1)] untypable (n = 2), followed by CC8 [(n = 31), STs; 8 (n = 29), 9003 (n = 2)] and CC93 (n = 3, ST 93) (Figs 4 and 5).

The *mecA* gene was detected in seventy-five out of eighty isolates. The five isolates lacking the *mecA* gene were all part of the CC5 clade and were isolated from sterile sites. Clades were evenly distributed across geographic regions, including America (blue tones), Europe (green tones), and Asia (red tones), as well as across different isolation years. The most recent similar genomes were from Asia (China, 2023, GCF_037911155.1; Taiwan, 2022, GCF_032459935.1) and North America (United States, 2021, GCF_038096265.1). These isolates belonged to the CC8, were isolated from sterile sites, and harbored the *mecA* gene.

When examining phylogenetic distances (Fig 5) the distance between the CC93 clade and the CC5 and CC8 clades was 0.008 (one mismatch every 125 nucleotides), while the distance between CC5 and CC8 was approximately 0.005 (one mismatch every 200 nucleotides) between them.

## Discussion

The molecular epidemiology of *S. aureus*, including associated antibiotic resistance and virulence factors, change by geographical location. Thus, molecular surveillance for clinically relevant MRSA isolates is key, and must be performed in different populations. In our study, we determined the genomic characteristics of 27 MRSA strains collected in 2022 and 2023 from eight centers in Mexico.

Herein, the most frequent ST found was ST1011, followed by ST5. Both STs had been previously reported in Mexico as a divergent subclade of CC-II A, whose early-branching strains were from the United States and whose most recent common ancestor was estimated to date from the late 1990s [24].

The replacement of CC30 by CC5 was described in Mexico in 2001 [25]. Challagundia et al., previous work on CC5 includes prevalent clones in the Western Hemisphere whose expansion revealed genomic changes toward increased antibiotic resistance and decreased virulence, as shown by the loss of the staphylococcal enterotoxin p (*sep*) gene from the immune evasion gene cluster of phage ФSa3, which was also absent from the studied MRSA strains [24].

Several studies have reported the molecular characteristics of MRSA isolates in Mexico, including an analysis of 249 *S. aureus* (MRSA and MSSA) blood isolates collected from pediatric patients at a tertiary care hospital from 2006 to 2019. In this study, 38% of isolates were MRSA, and SCC*mec-*II was the most frequently detected element (86.3%) [26], as reported in our study (*n* = 17, 63%). Additionally, the *pvl* gene was detected in four MRSA SCC*mec* IV isolates, in which most SCC*mec* IVa strains harbored the *pvl* genes.

In another study in Mexico, the molecular characteristics of 51 MRSA isolates collected from a tertiary care hospital between 2017 and 2018 were analyzed. Researchers reported that, most strains harbored the SCC*mec* type II (76.4%), with ST5-MRSA-II-t895 (New York/Japan clone) and ST1011-MRSA-II-t9364 (New York/Japan-Mexican variant clone). We should note that different lineages of CC5 (85.4%) and CC8 (8.3%) were identified in that study. This study is the first to report the association between the t895 and t9364 *spa* types and the ST5 and ST1011 lineages [27].

As for our work, we detected both clones with six strains being ST5(CC5)-MRSA-IIa-t895 (New York/Japan clone) and five being the ST1011(CC5)-MRSA-IIa, *t*9364 (New York/Japan-Mexican variant clone). Other relevant detected strains included ST1011(CC5)-MRSA-IIa, t895 (four strains), and ST8(CC8)-MRSA-IVa, *t*8 (three strains).

Interestingly, another study from Mexico reported that, of 107 MRSA strains collected from a third-level hospital in Mexico between 2009 and 2010, the most frequently detected SCC*mec* types were I and II and the ST247 and ST5 [28]. Furthermore, recent studies have documented the emergence of novel genetic lineages of MRSA in Latin America. Particularly, scientists have characterized bloodstream MRSA recovered from nine Latin American countries, including Mexico, with most MRSA isolates being CC5. In fact, CC5 has been shown to be the most MRSA in blood isolates from Latin America (except for Colombia and Ecuador) [29].

The MRSA molecular types reported for Mexico have also been reported for other countries. A molecular epidemiological analysis of MRSA strains isolated from blood in Japan was recently conducted, and this analysis encompassed 88 isolates. The prevalence of SCC*mec* type II was high in 2014 strains, accounting for a significant portion of the strains studied, however, its presence was significantly reduced in 2018 strains. Conversely, the prevalence of SCC*mec* type IV strains showed a marked increase, rising from 18.75% in 2014 to 83.87% in 2018. Furthermore, CC5, CC8, and CC1 were detected between 2015 and 2017, with ST1 being dominant [30].

A recent review included reports on the molecular characterization of clinical *S. aureus* from Malaysia from 2008 to 2020, in which hospital-acquired MRSA (HA-MRSA) and community-acquired MRSA (CA-MRSA) were included. Among HA-MRSA, the ST22-t032-SCC*mec* IV MRSA clone was reported to supplant the previous dominating clone, ST239-t037-SCC*mec* III. Meanwhile, ST30, ST772, ST6, and ST22 were repeatedly detected in CA-MRSA, with none being dominant [31].

An Australian report analyzing 620 MRSA samples collected from three hospitals (two adult and one pediatric) between 2017 and 2021 found that ST93 was the most common strain, corroborating previous Australian studies [32,33]. Our study detected ST5, similar to previous research, and additional STs including ST30, ST72, ST78, ST88, ST93, ST97, and ST239 [34].

In addition, a report examined the molecular characteristics of *S. aureus* isolated from China in 2021 and 2022, including both MRSA and MSSA. Forty STs were found in 214 clinical *S. aureus* isolates, with ST59 and ST6697 displaying the most extensive distribution and a higher CC5 frequency [35].

It has been reported that MRSA epidemics have occurred in waves, with fitter and more adaptable lineages replacing previously successful lineages. It is expected that variations may occur across the globe and are associated with different selection pressures that result in epidemiological differences by geographic region. While MRSA possesses a diverse arsenal of toxins, the success of a lineage involves more than just producing toxins but also the acquisition or loss of genetic elements involved in the colonization and adaptation of the strains [5].

The RefSeq database provided information about the global diversity of *S. aureus* strains, by the inclusion of 53 genomes that combined with our strains give 80 isolates distributed all over the world, including countries from America, Europe, and Asia. In all these strains, CC5 was the most frequent (*n* = 46), followed by CC8 (*n* = 31). As expected, the isolates in this study grouped with those from North and South America, predominantly from 2010 to 2015. Interestingly, the most recent similar genomes were from China and the United States, underlying the importance of the high mobility of populations in the present years.

MRSA strains typically harbor the *mecA* gene, which encodes Penicillin-Binding Protein 2a (PBP2a). This protein is crucial for resistance to methicillin and other extended-spectrum β-lactam antibiotics in MRSA [36]. Previous reports have identified MRSA *mecA* negative strains that possess a genetic marker, termed *mecC*, which encodes a trans-peptidase exhibiting a 63% similarity to PBP2a. *mecC*-containing MRSA strains are often associated with cattle and other animals and can be transferred to human MRSA populations [37]. In our study, the *mecA* gene was detected in 75 out of the 80 isolates selected from the RefSeq assembly database, and the five isolates lack the *mecA* gene. All these five strains (CC5 clade) were isolated from sterile sites from humans and are among the genomes retrieved (accession numbers: GCF 030515335.1; GCF 030512085.1; GCF 025505995.1; GCF 022326745.1; GCF 000609765.1).

Our research involved comparing the sequenced isolates to publicly available genomes using multiple bioinformatic tools. The genomes exhibiting the greatest similarity were identified, leading to the selection of a final sample set comprised of 80 isolates representing the most closely related genomes.

In this study, all centers from the Invifar network were invited to participate and only twenty-seven clinical isolates were recovered from sterile fluids in one year; Thus, these strains may not represent the circulating strains in Mexico. All twenty-seven clinical isolates were further analyzed by WGS, with no selection of strains performed. We decided to make this report because there is a lack of information about the clinically relevant circulating clinical isolates analyzed by WGS in Mexico, as noted by the reported MRSA genomes in the https://pubmlst.org/ database, in which only six strains recovered from milk from Mexico were deposited (revised on November 27, 2024). The use of WGS will allow other researchers to continue with analysis of genomes and overall, compare them with other genomes deposited all around the world. Other MRSA clinical isolates were recovered but these strains were collected from sites where normal microbiota may be present; Thus, the clinical relevance of the isolates may be questionable.

This study has several limitations. First, we only included isolates from eight centers in Mexico; thus, differences may be observed in other hospitals or geographic areas of the country. Second, we did not collect any clinical information on patients; thus, no classification of HA-MRSA or CA-MRSA was performed; and third, only 27 strains were included in the study. Hence, it should be noted that the 27 strains represent all strains collected over two years from sterile sites in eight centers.

In conclusion, the most frequent MRSA ST detected was ST1011, followed by ST5. Moreover, the SCC*mec* elements detected were SCC*mec* type IIa, followed by type IVa. High MIC values were observed for ciprofloxacin, erythromycin, and clindamycin, particularly within SCC*mec* IIa. Among the SCC*mec* IVa strains, most harbored the *lukS*-PV and *lukF*-PV genes.

## Supporting information

S1 Table Genome assembly quality metrics. Quality metrics were obtained from the PathogenWatch platform, genome size and %GC content is consistent with *S. aureus* genomic characteristics.
(DOCX)

S2 Table RefSeq assemblies included for comparison. Metadata for the included assemblies was obtained using the NCBI toolkit command-line tool.
(DOCX)

## References

[1] Velázquez-Meza ME. *Staphylococcus aureus* methicillin-resistant: emergence and dissemination. Salud Publica Mex. 2005;47(5):381–7. doi: 10.1590/s0036-36342005000500009 16323532

[2] Shallcross LJ, Fragaszy E, Johnson AM, Hayward AC. The role of the Panton-Valentine leucocidin toxin in staphylococcal disease: a systematic review and meta-analysis. Lancet Infect Dis. 2013;13(1):43–54. doi: 10.1016/S1473-3099(12)70238-4 23103172 PMC3530297

[3] Campbell AJ, Yazidi LSA, Phuong LK, Leung C, Best EJ, Webb RH, et al. Pediatric *Staphylococcus aureus* bacteremia: clinical spectrum and predictors of poor outcome. Clin Infect Dis. 2022;74(4):604–13. doi: 10.1093/cid/ciab510 34089594

[4] Lakhundi S, Zhang K. Methicillin-resistant *Staphylococcus aureus*: molecular characterization, evolution, and epidemiology. Clin Microbiol Rev. 2018;31(4):e00020-18. doi: 10.1128/CMR.00020-18 30209034 PMC6148192

[5] Jiang J-H, Cameron DR, Nethercott C, Aires-de-Sousa M, Peleg AY. Virulence attributes of successful methicillin-resistant Staphylococcus aureus lineages. Clin Microbiol Rev. 2023;36(4):e0014822. doi: 10.1128/cmr.00148-22 37982596 PMC10732075

[6] Hurley JC. Risk of death from methicillin-resistant *Staphylococcus aureus* bacteraemia: a meta-analysis. Med J Aust. 2002;176(4):188; author reply 189. doi: 10.5694/j.1326-5377.2002.tb04355.x 11913923

[7] Guo Y, Song G, Sun M, Wang J, Wang Y. Prevalence and therapies of antibiotic-resistance in *Staphylococcus aureus*. Front Cell Infect Microbiol. 2020;10:107. doi: 10.3389/fcimb.2020.00107 32257966 PMC7089872

[8] Katayama Y, Ito T, Hiramatsu K. A new class of genetic element, staphylococcus cassette chromosome mec, encodes methicillin resistance in *Staphylococcus aureus*. Antimicrob Agents Chemother. 2000;44(6):1549–55. doi: 10.1128/AAC.44.6.1549-1555.2000 10817707 PMC89911

[9] Bens CCPM, Voss A, Klaassen CHW. Presence of a novel DNA methylation enzyme in methicillin-resistant *Staphylococcus aureus* isolates associated with pig farming leads to uninterpretable results in standard pulsed-field gel electrophoresis analysis. J Clin Microbiol. 2006;44(5):1875–6. doi: 10.1128/JCM.44.5.1875-1876.2006 16672428 PMC1479204

[10] Kumar S, Anwer R, Yadav M, Sehrawat N, Singh M, Kumar V. Molecular typing and global epidemiology of Staphylococcus aureus. Current Pharmacol Rep. 2021;7(5):179–86. doi: 10.1007/s40495-021-00264-7

[11] Sawa T, Kooguchi K, Moriyama K. Molecular diversity of extended-spectrum β-lactamases and carbapenemases, and antimicrobial resistance. J Intensive Care. 2020;8:13. doi: 10.1186/s40560-020-0429-6 32015881 PMC6988205

[12] Humphreys H, Coleman DC. Contribution of whole-genome sequencing to understanding of the epidemiology and control of meticillin-resistant *Staphylococcus aureus*. J Hosp Infect. 2019;102(2):189–99. doi: 10.1016/j.jhin.2019.01.025 30721732

[13] Sabat AJ, Budimir A, Nashev D, Sá-Leão R, van Dijl JM, Laurent F, et al. Overview of molecular typing methods for outbreak detection and epidemiological surveillance. Euro Surveill. 2013;18(4):20380. doi: 10.2807/ese.18.04.20380-en 23369389

[14] Laupland KB, Lyytikäinen O, Søgaard M, Kennedy KJ, Knudsen JD, Ostergaard C, et al. The changing epidemiology of *Staphylococcus aureus* bloodstream infection: a multinational population-based surveillance study. Clin Microbiol Infect. 2013;19(5):465–71. doi: 10.1111/j.1469-0691.2012.03903.x 22616816

[15] CLSI, M100-S32. Performance standards for antimicrobial susceptibility testing; twenty-second informational supplement. Wayne, PA: Clinical and Laboratory Standars Institute; 2022.

[16] Pathogenwatch A. Global platform for genomic surveillance. 2019.

[17] Pierce NT, Irber L, Reiter T, Brooks P, Brown CT. Large-scale sequence comparisons with sourmash. F1000Res. 2019;8:1006. doi: 10.12688/f1000research.19675.1 31508216 PMC6720031

[18] Seemann T. Prokka: rapid prokaryotic genome annotation. Bioinformatics. 2014;30(14):2068–9. doi: 10.1093/bioinformatics/btu153 24642063

[19] Page AJ, Cummins CA, Hunt M, Wong VK, Reuter S, Holden MTG, et al. Roary: rapid large-scale prokaryote pan genome analysis. Bioinformatics. 2015;31(22):3691–3. doi: 10.1093/bioinformatics/btv421 26198102 PMC4817141

[20] Löytynoja A. Phylogeny-aware alignment with PRANK. Methods Mol Biol. 2014;1079:155–70. doi: 10.1007/978-1-62703-646-7_10 24170401

[21] Stamatakis A. RAxML version 8: a tool for phylogenetic analysis and post-analysis of large phylogenies. Bioinformatics. 2014;30(9):1312–3. doi: 10.1093/bioinformatics/btu033 24451623 PMC3998144

[22] Camacho C, Coulouris G, Avagyan V, Ma N, Papadopoulos J, Bealer K, et al. BLAST+: architecture and applications. BMC Bioinformatics. 2009;10:421. doi: 10.1186/1471-2105-10-421 20003500 PMC2803857

[23] Letunic IA-O, Bork P. Interactive Tree Of Life (iTOL) v5: an online tool for phylogenetic tree display and annotation. (1362-4962 (Electronic)). 10.1093/nar/gkab301 PMC8265157 33885785

[24] Challagundla L, Reyes J, Rafiqullah I, Sordelli DO, Echaniz-Aviles G, Velazquez-Meza ME, et al. Phylogenomic classification and the evolution of clonal complex 5 methicillin-resistant. Front Microbiol. 2018;9:1901. doi: 10.3389/fmicb.2018.01901 30186248 PMC6113392

[25] Aires De Sousa M, Miragaia M, Sanches IS, Avila S, Adamson I, Casagrande ST, et al. Three-year assessment of methicillin-resistant Staphylococcus aureus clones in Latin America from 1996 to 1998. J Clin Microbiol. 2001;39(6):2197–205. doi: 10.1128/JCM.39.6.2197-2205.2001 11376057 PMC88111

[26] Vazquez-Rosas GJ, Merida-Vieyra J, Aparicio-Ozores G, Lara-Hernandez A, De Colsa A, Aquino-Andrade A. Molecular characterization of *Staphylococcus aureus* obtained from blood cultures of paediatric patients treated in a Tertiary Care Hospital in Mexico. Infect Drug Resist. 2021;14:1545–56. doi: 10.2147/IDR.S302416 33911882 PMC8071697

[27] Negrete-González C, Turrubiartes-Martínez E, Galicia-Cruz OG, Noyola DE, Martínez-Aguilar G, Pérez-González LF, et al. High prevalence of t895 and t9364 spa types of methicillin-resistant *Staphylococcus aureus* in a tertiary-care hospital in Mexico: different lineages of clonal complex 5. BMC Microbiol. 2020;20(1):213. doi: 10.1186/s12866-020-01881-w 32689948 PMC7370520

[28] Ortíz-Gil MÁ, Velazquez-Meza ME, Echániz-Aviles G, Mora-Domínguez JP, Carnalla-Barajas MN, Mendiola Del Moral EL. Tracking methicillin-resistant *Staphylococcus aureus* clones in a hospital in Southern Mexico. Salud Publica Mex. 2020;62(2):186–91. doi: 10.21149/10786 32237561

[29] Arias C, Reyes J, Carvajal LP, Rincon S, Diaz L, Panesso D, et al. A prospective cohort multicenter study of molecular epidemiology and phylogenomics of *Staphylococcus aureus* bacteremia in nine Latin American countries. Antimicrob Agents Chemother. 2017;61(10):e00816–17. doi: 10.1128/AAC.00816-17 28760895 PMC5610503

[30] Sato T, Yamaguchi T, Aoki K, Kajiwara C, Kimura S, Maeda T, et al. Whole-genome sequencing analysis of molecular epidemiology and silent transmissions causing meticillin-resistant *Staphylococcus aureus* bloodstream infections in a university hospital. J Hosp Infect. 2023;139:141–9. doi: 10.1016/j.jhin.2023.05.014 37301229

[31] Junaidi NSSA, Mohamed Shakrin NNS, Mohd Desa MN, Wan Yunus WMZ. Dissemination pattern of hospital-acquired methicillin-resistant. Malays J Med Sci. 2023;30(2):26–41. doi: 10.21315/mjms2023.30.2.3 37102054 PMC10125240

[32] Munckhof WJ, Schooneveldt J, Coombs GW, Hoare J, Nimmo GR. Emergence of community-acquired methicillin-resistant *Staphylococcus aureus* (MRSA) infection in Queensland, Australia. Int J Infect Dis. 2003;7(4):259–64. doi: 10.1016/s1201-9712(03)90104-4 14656416

[33] Chua KYL, Seemann T, Harrison PF, Monagle S, Korman TM, Johnson PDR, et al. The dominant Australian community-acquired methicillin-resistant Staphylococcus aureus clone ST93-IV [2B] is highly virulent and genetically distinct. PLoS ONE. 2011;6(10):e25887. doi: 10.1371/journal.pone.0025887 21991381 PMC3185049

[34] Forde BM, Bergh H, Cuddihy T, Hajkowicz K, Hurst T, Playford EG, et al. Clinical implementation of routine whole-genome sequencing for hospital infection control of multi-drug resistant pathogens. Clin Infect Dis. 2023;76(3):e1277–84. doi: 10.1093/cid/ciac726 36056896

[35] Zhang H, Cao J, He Z, Zong X, Sun B. Molecular epidemiology of *Staphylococcus aureus* in a Tertiary Hospital in Anhui, China: ST59 remains a serious threat. Infect Drug Resist. 2023;16:961–76. doi: 10.2147/IDR.S395220 36814828 PMC9940498

[36] Kim C, Milheiriço C, Gardete S, Holmes MA, Holden MTG, de Lencastre H, et al. Properties of a novel PBP2A protein homolog from Staphylococcus aureus strain LGA251 and its contribution to the β-lactam-resistant phenotype. J Biol Chem. 2012;287(44):36854–63. doi: 10.1074/jbc.M112.395962 22977239 PMC3481288

[37] Vandendriessche S, Vanderhaeghen W, Soares FV, Hallin M, Catry B, Hermans K, et al. Prevalence, risk factors and genetic diversity of methicillin-resistant Staphylococcus aureus carried by humans and animals across livestock production sectors. J Antimicrob Chemother. 2013;68(7):1510–6. doi: 10.1093/jac/dkt047 23429641

