# Supplementary material for: Genomic characteristics and molecular epidemiology of MRSA from medical centers in Mexico: Results from the Invifar network
Source: PLoS One. 2025 Jan 27;20(1):e0317284. doi: 10.1371/journal.pone.0317284 (PMC11771916; doi:10.1371/journal.pone.0317284)
Supplement: S1 Table — Quality metrics were obtained from the PathogenWatch platform, genome size and %GC content is consistent with S. aureus genomic characteristics. (DOCX) [file pone.0317284.s001.docx]

**S1 Table.** **Genome assembly quality metrics**. Quality metrics were obtained from the PathogenWatch platform, genome size and %GC content is consistent with *S. aureus* genomic characteristics.

| Strain | Genome size (bp) | Contig N50 | Contigs | % GC |
| --- | --- | --- | --- | --- |
| A23-2905 | 2801846 | 222016 | 31 | 32.7 |
| A23-2388 | 2880143 | 235664 | 38 | 32.7 |
| A23-2539 | 2792910 | 243850 | 34 | 32.7 |
| A23-2580 | 2761822 | 182523 | 39 | 32.7 |
| A22-1364 | 2827559 | 243848 | 46 | 32.8 |
| A22-1243 | 2841448 | 589160 | 38 | 32.6 |
| A22-2959 | 2883971 | 204789 | 42 | 32.7 |
| A23-2574 | 2881321 | 235658 | 41 | 32.7 |
| A22-1697 | 2845744 | 174131 | 35 | 32.7 |
| A23-2538 | 2839032 | 222522 | 40 | 32.7 |
| A23-2515 | 2781122 | 729674 | 22 | 32.7 |
| A23-2165 | 2795758 | 260868 | 35 | 32.7 |
| A23-2228 | 2888104 | 589262 | 34 | 32.7 |
| A23-2905 | 2801846 | 222016 | 31 | 32.7 |
| A22-1714 | 2803818 | 222016 | 31 | 32.7 |
| A22-2940 | 2840164 | 222612 | 46 | 32.7 |
| A22-1695 | 2760998 | 297280 | 48 | 32.7 |
| A22-2979 | 2884707 | 222609 | 40 | 32.7 |
| A22-1752 | 2788989 | 222016 | 28 | 32.7 |
| A22-1232 | 2841883 | 589137 | 37 | 32.6 |
| A22-2540 | 2878588 | 235664 | 48 | 32.7 |
| A23-2537 | 2792694 | 243850 | 36 | 32.7 |
| A23-2229 | 2913360 | 201442 | 48 | 32.6 |
| A23-2619 | 2759845 | 181191 | 44 | 32.7 |
| A23-2511 | 2783092 | 589023 | 35 | 32.6 |
| A23-2201 | 2753554 | 243849 | 31 | 32.7 |
| A23-2321 | 2791646 | 182785 | 43 | 32.6 |
| A23-2575 | 2875196 | 154008 | 80 | 32.7 |
| A23-2438 | 2831035 | 194939 | 42 | 32.7 |
