# Supplementary material for: Genomic characteristics and molecular epidemiology of MRSA from medical centers in Mexico: Results from the Invifar network
Source: PLoS One. 2025 Jan 27;20(1):e0317284. doi: 10.1371/journal.pone.0317284 (PMC11771916; doi:10.1371/journal.pone.0317284)
Supplement: S2 Table — Metadata for the included assemblies was obtained using the NCBI toolkit command-line tool. (DOCX) [file pone.0317284.s002.docx]

**S2 Table. RefSeq assemblies included for comparison.** Metadata for the included assemblies was obtained using the NCBI toolkit command-line tool.

| Assembly accession | Assembly identifier | ST | Clonal complex | *mec*A | Country of isolation | Year of isolation | Isolated from sterile site |
| --- | --- | --- | --- | --- | --- | --- | --- |
| GCF_036421195.1 | A23_2165 | 93 | CC93 | Present | Mexico | 2023 | Yes |
| GCF_008762475.1 | GCF_008762475.1 | 93 | CC93 | Present | Australia | 2015 | No |
| GCF_900250335.1 | GCF_900250335.1 | 93 | CC93 | Present | Denmark | 2014 | Not specified |
| GCF_000560285.1 | GCF_000560285.1 | 8 | CC8 | Present | USA | 2011 | No |
| GCF_000695875.1 | GCF_000695875.1 | 8 | CC8 | Present | Belgium | 2012 | No |
| GCF_001207235.1 | GCF_001207235.1 | 8 | CC8 | Present | USA | 2011 | Yes |
| GCF_002123015.1 | GCF_002123015.1 | 8 | CC8 | Present | USA | 2015 | No |
| GCF_002125175.1 | GCF_002125175.1 | 8 | CC8 | Present | USA | 2011 | No |
| GCF_036421395.1 | A23_2511 | 8 | CC8 | Present | Mexico | 2023 | Yes |
| GCF_037911155.1 | GCF_037911155.1 | 8 | CC8 | Present | China | 2023 | Yes |
| GCF_014695075.1 | GCF_014695075.1 | 8 | CC8 | Present | USA | 2017 | No |
| GCF_012274765.1 | GCF_012274765.1 | 8 | CC8 | Present | USA | 2012 | No |
| GCF_038096265.1 | GCF_038096265.1 | 8 | CC8 | Present | USA | 2021 | Yes |
| GCF_001448185.1 | GCF_001448185.1 | 8 | CC8 | Present | Switzerland | 2013 | Yes |
| GCF_014181095.1 | GCF_014181095.1 | 8 | CC8 | Present | Germany | 2018 | Not specified |
| GCF_021852645.1 | GCF_021852645.1 | 8 | CC8 | Present | USA | 2017 | Yes |
| GCF_001352815.1 | GCF_001352815.1 | 8 | CC8 | Present | USA | 2009 | No |
| GCF_036421375.1 | A23_2228 | 8 | CC8 | Present | Mexico | 2023 | Yes |
| GCF_036421015.1 | A23_2229 | 8 | CC8 | Present | Mexico | 2023 | Yes |
| GCF_002000625.1 | GCF_002000625.1 | 8 | CC8 | Present | Suriname | 2013 | No |
| GCF_003571365.1 | GCF_003571365.1 | 8 | CC8 | Present | Suriname | 2012 | No |
| GCF_001206095.1 | GCF_001206095.1 | 8 | CC8 | Present | USA | 2010 | No |
| GCF_001354015.1 | GCF_001354015.1 | 8 | CC8 | Present | USA | 2009 | Yes |
| GCF_008364195.1 | GCF_008364195.1 | 8 | CC8 | Present | USA | 2019 | Not specified |
| GCF_001353795.1 | GCF_001353795.1 | 8 | CC8 | Present | USA | 2010 | No |
| GCF_001212305.1 | GCF_001212305.1 | 8 | CC8 | Present | USA | 2010 | Yes |
| GCF_000539075.1 | GCF_000539075.1 | 8 | CC8 | Present | USA | 2014 | Yes |
| GCF_036421275.1 | A22_1232 | 9003 | CC8 | Present | Mexico | 2023 | Yes |
| GCF_036421755.1 | A22_1243 | 9003 | CC8 | Present | Mexico | 2023 | Yes |
| GCF_032459935.1 | GCF_032459935.1 | 8 | CC8 | Present | Taiwan | 2022 | Yes |
| GCF_003073435.1 | GCF_003073435.1 | 8 | CC8 | Present | Spain | 2015 | No |
| GCF_000571735.1 | GCF_000571735.1 | 8 | CC8 | Present | USA | 2012 | No |
| GCF_002088995.1 | GCF_002088995.1 | 8 | CC8 | Present | Canada | 2004 | No |
| GCF_029809195.1 | GCF_029809195.1 | 8 | CC8 | Present | Taiwan | 2010 | Yes |
| GCF_030758795.1 | GCF_030758795.1 |  |  | Present | USA | 2018 | Not specified |
| GCF_030515335.1 | GCF_030515335.1 | 5 | CC5 | Absent | USA | 2016 | Yes |
| GCF_030512085.1 | GCF_030512085.1 | 5 | CC5 | Absent | USA | 2011 | Yes |
| GCF_036421455.1 | A23_2321 | 9034 | CC5 | Present | Mexico | 2023 | Yes |
| GCF_036421495.1 | A23_2619 | 9034 | CC5 | Present | Mexico | 2023 | Yes |
| GCF_036421695.1 | A23_2580 | 9034 | CC5 | Present | Mexico | 2023 | Yes |
| GCF_000595005.1 | GCF_000595005.1 | 5 | CC5 | Present | USA | 1991 | Not specified |
| GCF_025505995.1 | GCF_025505995.1 | 5 | CC5 | Absent | France | 2006 | Yes |
| GCF_022326745.1 | GCF_022326745.1 | 5 | CC5 | Absent | USA | 2010 | No |
| GCF_036421075.1 | A22_1695 | 5 | CC5 | Present | Mexico | 2022 | Yes |
| GCF_002260225.1 | GCF_002260225.1 | 5 | CC5 | Present | Guatemala | 2012 | Yes |
| GCF_000609765.1 | GCF_000609765.1 | 5 | CC5 | Absent | USA | 2007 | Yes |
| GCF_003237275.1 | GCF_003237275.1 |  |  | Present | Italy | 2015 | No |
| GCF_003813355.1 | GCF_003813355.1 | 5 | CC5 | Present | Australia | 2015 | No |
| GCF_003720235.1 | GCF_003720235.1 | 225 | CC5 | Present | USA | 2012 | No |
| GCF_036533395.1 | GCF_036533395.1 | 105 | CC5 | Present | USA | 2020 | No |
| GCF_036632495.1 | GCF_036632495.1 | 105 | CC5 | Present | USA | 2010 | No |
| GCF_002120975.1 | GCF_002120975.1 | 5 | CC5 | Present | USA | 2016 | No |
| GCF_036632555.1 | GCF_036632555.1 | 5 | CC5 | Present | USA | 2017 | No |
| GCF_000360505.1 | GCF_000360505.1 | 5 | CC5 | Present | USA | 2004 | Not specified |
| GCF_000553675.1 | GCF_000553675.1 | 5 | CC5 | Present | USA | 2003 | No |
| GCF_000546865.1 | GCF_000546865.1 | 5 | CC5 | Present | USA | 2004 | Not specified |
| GCF_000557605.1 | GCF_000557605.1 | 5 | CC5 | Present | USA | 2004 | No |
| GCF_000544425.1 | GCF_000544425.1 | 5 | CC5 | Present | USA | 2004 | No |
| GCF_000552785.1 | GCF_000552785.1 | 5 | CC5 | Present | USA | 2003 | No |
| GCF_000551445.1 | GCF_000551445.1 | 5 | CC5 | Present | USA | 2003 | Yes |
| GCF_000360125.1 | GCF_000360125.1 | 5 | CC5 | Present | USA | 2003 | Not specified |
| GCF_036421235.1 | A22_1714 | 5 | CC5 | Present | Mexico | 2022 | Yes |
| GCF_002267765.1 | GCF_002267765.1 | 5 | CC5 | Present | Mexico | 2012 | Yes |
| GCF_036421415.1 | A23_2438 | 5 | CC5 | Present | Mexico | 2023 | Yes |
| GCF_036421515.1 | A23_2905 | 5 | CC5 | Present | Mexico | 2023 | Yes |
| GCF_036421255.1 | A22_1697 | 5 | CC5 | Present | Mexico | 2022 | Yes |
| GCF_036421335.1 | A23_2959 | 5 | CC5 | Present | Mexico | 2023 | Yes |
| GCF_036421355.1 | A23_2979 | 5 | CC5 | Present | Mexico | 2023 | Yes |
| GCF_036421215.1 | A22_1752 | 1011 | CC5 | Present | Mexico | 2022 | Yes |
| GCF_036421315.1 | A23_2538 | 1011 | CC5 | Present | Mexico | 2023 | Yes |
| GCF_036421675.1 | A23_2537 | 1011 | CC5 | Present | Mexico | 2023 | Yes |
| GCF_036421735.1 | A23_2539 | 1011 | CC5 | Present | Mexico | 2023 | Yes |
| GCF_000605085.1 | GCF_000605085.1 | 1011 | CC5 | Present | USA | 2013 | No |
| GCF_036421575.1 | A23_2201 | 1011 | CC5 | Present | Mexico | 2023 | Yes |
| GCF_036421295.1 | A22_1364 | 1011 | CC5 | Present | Mexico | 2022 | Yes |
| GCF_036421535.1 | A23_2940 | 1011 | CC5 | Present | Mexico | 2023 | Yes |
| GCF_036421635.1 | A23_2574 | 1011 | CC5 | Present | Mexico | 2023 | Yes |
| GCF_036421655.1 | A23_2575 | 1011 | CC5 | Present | Mexico | 2023 | Yes |
| GCF_036421715.1 | A23_2540 | 1011 | CC5 | Present | Mexico | 2023 | Yes |
| GCF_036421435.1 | A23_2388 | 1011 | CC5 | Present | Mexico | 2023 | Yes |
